# Maternal stature, maternal education and child growth in Pakistan: a cross-sectional study

**DOI:** 10.3934/publichealth.2020032

**Published:** 2020-06-15

**Authors:** Nazli Javid, Christy Pu

**Affiliations:** Institute of Public Health, National Yang-Ming University, Taiwan

**Keywords:** maternal stature, maternal weight, stunting, children growth, Pakistan

## Abstract

Pakistan has a significantly higher prevalence of stunted children under five years old compared with other countries with a similar income level. Given maternal education is a modifiable factor, we analyzed whether education has a larger marginal effect on improving children's growth for shorter stature mothers. Pakistan Demographic and Health Survey of 2012–13 was analyzed, with a total of 3,883 of children under five years of age (belonged to 2,327 mothers). The results showed that the overall prevalence of stunting, underweight, wasting, and overweight in our sample was 45%, 26.2%, 9.9%, and 9.5%, respectively. Short stature mothers have a higher number of malnourished children as compared to taller mothers. Compared to tall stature mothers, short stature mothers at all education levels have a higher number of stunted and underweight children. Maternal education has a significant positive effect on children's growth. However, we did not find significant differences in the marginal effect of maternal education among mothers with different statures. Policies providing specialized care to children born to short stature mothers are crucial, along with emphasizing mothers' education. Moreover, a poverty elevation program is necessary as a significant fraction of childhood malnutrition is attributed to the wealth index.

## 1. Introduction

Despite various advancements and improvements in child health in the last decade, malnutrition is still a critical public health challenge in the 21st century [1]. Malnutrition affects children in four different ways: (i) stunted (low-height-for-age); (ii) underweight (low-weight-for-age); (iii) wasted (low weight for height), and (iv) overweight (high weight-for-height). Stunting at the age of two is considered as the best predictor of poor future human capital, and undesirable health outcomes can happen due to stunting before age five [2],[3]. It was estimated that in 2018, 21.9% (149 million) children were stunted worldwide, while 7.3% (49 million) and 5.9% (40 million) children were wasted and overweight, respectively [4]. More importantly, Asia and Africa are regions of a higher prevalence of children malnutrition. Pakistan, a South Asian country, has the highest growth rate of stunted children (45%), followed by Afghanistan (41%) [5]. Despite some progress in the health sectors, the higher prevalence of undernutrition children under age five is one of the main public health problems in Pakistan.

Children born to short stature mothers often face impaired growth in infancy [6]. According to the World Health Organization (WHO), a 19-year-old female with height less than −2 standard deviation (SD) (<150 cm) is considered to have short stature [7],[8]. Infants born to short stature mothers have higher risks of being stunted as compared to infants born to taller mothers [9]. In particular, children with low birth weight born to short stature mothers are at a higher risk of stunting and have a poor growth rate [10],[11]. The reason behind the reduced fetal growth of infants born to short stature mothers may be undernutrition or malnutrition [12]. Since short stature mothers may have lower health stock, the available nutrients to the fetus may be insufficient. This could result in intrauterine growth restriction and low birth weight, influencing babies' health [13].

Previous studies reported a strong positive association between child growth and maternal education [14],[15]. A recent review of child growth in 14 countries by WHO showed a strong relationship between the reduced likelihood of childhood stunting and higher maternal education. Specifically, ten years of maternal schooling has been suggested as a solution to prevent stunting in Malawi, Tanzania, and Zimbabwe [16]. Several reasons have been provided to explain this relationship. First, education enables mothers to make educated decisions during child upbringing [17]. Second, mothers with formal education are usually employed, which allows them to gain better access to food for their children [18]. Third, educated mothers have the ability to identify different types of illnesses quickly and seek better healthcare [15]. According to the Pakistan Demographic Health Survey (PDHS) 2012–13 [19], a significant fraction of women (52.2%) has no formal education in Pakistan, and this is mainly due to poverty, child labor, gender discrimination, harmful social norms, insecurity, and dangers on the way to school [20].

Limited studies have been conducted on the relationship between maternal stature and children's stature in Pakistan. Among the few studies that were conducted in Pakistan, Ali et al. [21] found that low parental education and economic status have a significant association with malnutrition. Khan et al. [22] found that mothers with no formal education were more likely to have wasted children. Rizvi et al. [23] found that in Pakistan, child stunting was mainly associated with poverty, low maternal education, and maternal empowerment. None of these studies, however, cover all four indicators of malnutrition (stunting, underweight, wasting, and overweight).

Maternal nutrition plays a crucial role in the development of fetal growth. According to the Developmental Origins of Health and Disease (DOHaD) theory, adverse nutritional settings in pregnancy may change the structure and function of particular organs in the children permanently, which could lead to many diseases in adulthood [24]. By the concept of the DOHaD, the worsened health outcomes in adulthood is closely linked to undernutrition (stunting and wasting) and over-nutrition (overweight and obesity) in childhood [24]. This necessitates the prevention of adult disease through the promotion of maternal and child health and reducing malnutrition by providing nutritious foods, a well-balanced diet, and developing health-promoting policies to diminish the latent risks of diseases [25].

Since maternal stature may be relatively difficult to modify in the short run, we investigate whether maternal education act as a solution to improve children's stature given short maternal stature. The effect of maternal education by maternal stature has rarely been investigated by previous studies. We hypothesize that short stature mothers are more likely to have stunted or other presentations (wasted, underweight, and overweight/obesity) of children in Pakistan. More specifically, we hypothesize that babies born to tall stature mothers may already be at a lower risk of poor stature, additional maternal education may only have small marginal effects on further stature improvement. In contrast, babies born to shorter mothers may have more room for improvement and hence make the marginal effect of maternal education larger.

The objectives of this study are: (1) To assess the prevalence of stunting, underweight, overweight, and wasting using actual measured weights and heights of children born to short stature mothers (height < 150 cm) and tall stature mothers (height ≥ 150 cm) in Pakistan. (2) To study the relationship between these anthropometric measurements and selected sociodemographic characteristics such as maternal age, weight, education, socioeconomic status, child age, child sex, and area of residence (rural and urban). (3) To determine whether maternal education is associated with growth for children born to mothers with different statures.

## 2. Methods

### 2.1. Demographic health survey

Demographic and Health Surveys (DHS) [26] is a project conducted by the United States Agency for International Development [27] with the technical assistance of Inner City Fund (ICF) International for conducting worldwide DHS surveys. The main aim of the DHS project is to help policymakers in their decisions by providing the most representative and accurate information about the population [28]. DHS are nationally represented with high-quality data and have been used frequently in nutritional and public health studies [21],[29]. It collects cross-sectional information on detailed demographic and health behavior variables and is repeated every five years. The data are publicly available [27].

We used the data of PDHS 2012–13 in this study. The PDHS 2012–13 was carried out by the National Institute of Population Studies, Pakistan [30]. The year 2012–13 was used because it was the most recent available year of the survey at the point of the study. A cross-sectional household survey was carried out to represent Pakistan using the multistage cluster sampling method, explained below.

#### 2.1.1. Sample design

The sample selection of the survey is based on the 1998 population census and covers all urban and rural areas. The survey consisted of two stages: (i) in the first stage; all cities were divided into enumeration blocks (small areas). There were 200–250 households in each block, on average. Based on the income levels, each block is further grouped into three categories (low, middle, and high).

#### 2.1.2. Household selection

In the second stage, a sample size of 14,000 households was estimated to provide rational precision for the survey indicators, which includes 248 urban and 250 rural survey sample areas across the country. Then sampling was accomplished where for each sampling point, 28 households were carefully chosen by applying a logical sampling technique with a random start for the selection of households. Throughout the country, a total of 498 areas with 14,000 households were selected for the survey, where 6,944 households were taken from the urban areas, and 7,056 households were included from the rural areas.

#### 2.1.3. Questionnaires

The survey of 2012–13 used four classes of questionnaires: (i) Household, (ii) Women, (iii) Men, and (iv) Community. The survey followed the standard model questionnaires for the DHS program. However, the questionnaires were accustomed wherever necessary to adjust to a country's values and norms. The National Institute of Population Studies consulted national and international experts via a series of meetings. As respondents may not be native English speakers, all questionnaires were rewritten into local languages according to the survey regions, approved by the technical committee. The surveys collected social demographic and socioeconomic variables of ever-married women and consists of data on the anthropometry measurements for children under age five.

In 2006, the World Health Organization introduced a new standard to measure child growth for children under age five globally [31]. Since 2006, the same standard of WHO has been used around the globe and serves a public ground for the analysis of child growth. According to this standard, stunting, wasting, underweight, and overweight are defined as follows:

- Stunting: children whose height for age < −2 SD of the WHO Child Growth Standards median.
- Wasting: children whose weight for height < −2 SD of the WHO Child Growth Standards median.
- Underweight: children whose weight for age < −2 SD of the WHO Child Growth Standards median.
- Overweight: children whose weight for height > +2 SD of the WHO Child Growth Standards median.

For the PDHS 2012–13, each interview team used a scale and a measurement board to measure children's weights and heights. The weights were measured using lightweight scales with digital screens designed and manufactured under the authority of the United Nations International Children's Fund Emergency Fund. The heights were measured via specially made measuring board. Children below age two or less than 85 cm were measured in lying down position on the board. All other children heights were measured on standing positions.

### 2.2. Study sample

There were a total of 3,883 children in the PDHS 2012–13. We excluded all those children (flagged values) whose height-for-age (HAZ) values fell outside ± 6 SD. Similar exclusion were used for weight-for-age (WAZ) (−6 SD below or +5 SD above), weight-for-height (WHZ) (±5 SD), and body mass index-for-age (BAZ) (±5 SD). These extreme values were excluded as they are most likely a result of errors in measurement or data entry [31]. This leaves us with a final sample of 3,354 children belonging to 2,327 mothers.

### 2.3. Statistical analysis

Mothers' sociodemographic characteristics were grouped by maternal stature < 150 cm and ≥ 150 cm and described using counts and percentages for categorical variables; means, and standard deviations for continuous variables. We report the prevalence of stunting, underweight, wasting, and overweight in percentages, and 95% CI. We used four separate logistic regression models to estimate risk factors associated with child physical status (i.e., stunting, underweight, wasting, and overweight) with (i) maternal (height, education, age, and weight), (ii) household (residence and wealth index or socioeconomic status), and (iii) child (sex and age) level variables.

We used a model-building strategy to formulate our models. We first conduct an unadjusted exploratory analysis to determine variables associated with each outcome. These variables must also be scientifically relevant to the outcome of children's stature. We then retain variables with a statistical significance of p < 0.25 based on the bivariate analysis. Wald tests for multiple coefficients are then applied to remove redundant variables to make the models parsimonious [32]. For all models, we retained the variable maternal weight and height, and maternal education regardless of statistical significance as these are the main variables of interest. For comparative purposes, we retain a variable for all four models once it is selected into any model. All statistical analyses were conducted using STATA MP Environment (StataCorp. 2017. *Stata Statistical Software: Release 15.* College Station, TX: StataCorp LLC.).

## 3. Results

In PDHS 2012–13, 13,944 households were selected for an interview, of which 12,943 completed the survey. There were 14,569 ever-married women of age 15–49 years in these households, and of which 13,558 were effectively questioned (93% response rate). Table 1 presents the sociodemographic factors of short (< 150 cm) and tall (≥ 150 cm) stature mothers. The mean age, weight, and height of short stature mothers were 29.1 years, 52.0 kg, and 145.0 cm, while tall stature mothers have the corresponding values of 29.8 years, 56.4 kg, and 156.5 cm, respectively. Compared to tall stature mothers, short stature mothers have lower education. The percentage of their children's sex are approximately equal. The anthropometry measurements were divided into five groups, each of length 12 months. We found that compared to tall mothers, short mothers have a higher prevalence of stunted and underweight children in each year-wise group, while wasting and overweight prevalence are different in each year-wise group. All the prevalence measurements were reported with 95% confidence intervals, wherever applicable.

**Table 1.** Sociodemographic factors of short and tall stature mothers with baseline characteristics of infants' anthropometry by maternal stature.

Sociodemographic factors	Maternal stature < 150 cm (n = 444)	Maternal stature ≥ 150 cm (n = 1883)	P value
Maternal age (years): mean (SD)	29.1 (6.6)	29.8 (6.4)	0.091
Maternal weight (kg): mean (SD)	52.0 (12.6)	58.6 (12.8)	<0.001
Maternal height (cm): mean (SD)	145.0 (9.9)	156.5 (4.8)	<0.001
Maternal education			<0.001
No education	60.1%	50.3%	
Primary	15.5%	15.7%	
Secondary	18.0%	20.2%	
Higher	6.3%	13.8%	
Child sex			0.311
Boys	312/636 (49.1%)	1394/2718 (51.3%)	
Girls	324/636 (50.9%)	1324/2718 (48.7%)	
Height (cm), weight (kg): (mean, SD)			
0–11 months	(60.9, 8.4), (6.1, 1.8)	(63.3, 9.2), (6.6, 1.9)	0.007, 0.002
12–23 months	(72.2, 8.1), (8.6, 1.6)	(75.6, 6.2), (9.1, 1.6)	<0.001, 0.006
24–35 months	(81.1, 8.4), (10.7, 1.9)	(83.3, 7.5), (11.2, 1.9)	0.011, 0.050
36–47 months	(85.5, 9.4), (12.2, 2.4)	(90.9, 8.5), (13.1, 2.1)	<0.001, <0.001
48–59 months	(91.6, 12.5), (14.1, 2.8)	(97.4, 9.5), (14.5, 2.3)	0.001, 0.111
Infant anthropometry			
Stunted(%)			
0–11 months	49/134, 36.6%	117/514, 22.8%	<0.001
12–23 months	74/108, 68.5%	187/484, 38.6%	
24–35 months	86/130, 66.2%	299/570, 52.5%	
36–47 months	93/128, 72.7%	264/563, 46.9%	
48–59 months	98/136, 72.1%	268/587, 445.7%	
Underweight(%)			
0–11 months	44/134, 32.8%	105/514, 20.4%	<0.001
12–23 months	45/108, 41.7%	116/484, 24.0%	
24–35 months	45/130, 34.6%	150/570, 26.3%	
36–47 months	50/128, 39.1%	134/563, 23.8%	
48–59 months	55/136, 40.4%	150/587, 25.6%	
Wasting(%)			
0–11 months	22/134, 16.4%	67/514, 13.0%	0.613
12–23 months	18/108, 16.7%	70/484, 14.5%	
24–35 months	8/130, 6.2%	46/570, 8.1%	
36–47 months	13/128 10.2%	40/563, 7.1%	
48–59 months	5/136, 3.7%	41/587, 7.0%	
Overweight(%)			
0–11 months	13/134, 9.7%	47/514, 9.1%	<0.001
12–23 months	16/108, 14.8%	27/484, 5.6%	
24–35 months	20/130, 15.4%	52/570, 9.1%	
36–47 months	22/128 17.2%	50/563, 8.9%	
48–59 months	27/136, 19.9%	47/587, 8.0%	

**Table 2.** Descriptive analysis of the prevalence of basic maternal, household, and child characteristics.

	N	Stunting	Wasting	Underweight	Overweight	*p_s_*	*p_w_*	*p_u_*	*p_o_*
Mother's education						<0.001	0.005	<0.001	<0.001
No education	1770	977 (55.2%)	206 (11.6%)	593 (33.5%)	186 (10.5%)				
Primary	518	240 (46.3%)	42 (8.1%)	138 (26.6%)	41 (7.9%)				
Secondary	673	206 (30.6%)	54 (8.0%)	115 (17.1%)	56 (8.3%)				
Higher	392	112 (28.5%)	28 (7.1%)	48 (12.2%)	38 (9.7%)				
Residence						<0.001	0.2745	0.2745	0.118
Urban	1471	598 (40.7%)	136 (9.2%)	319 (21.5%)	154 (10.5%)				
Rural	1833	937 (49.8%)	194 (10.3%)	575 (30.5%)	167 (8.9%)				
Maternal height						<0.001	0.485	<0.001	<0.001
<145 cm	165	120 (72.7%)	15 (9.1%)	72 (43.6%)	30 (18.2%)				
145–149.9 cm	492	293 (59.6%)	52 (10.6%)	173 (35.2%)	70 (14.2%)				
150–154.9 cm	1089	515 (47.3%)	117 (10.7%)	305 (28.0%)	94 (8.6%)				
≥155 cm	1608	607 (37.7%)	146 (9.1%)	344 (21.4 %)	127 (7.9%)				
Wealth index						<0.001	0.008	<0.001	<0.001
Poorest	688	427 (62.1%)	83 (12.1%)	256 (37.2%)	102 (14.8%)				
Poorer	659	360 (54.0%)	67 (10.2%)	215 (32.6%)	58 (8.8%)				
Middle	619	286 (46.2%)	65 (10.5%)	160 (25.8%)	52 (8.4%)				
Richer	706	278 (39.4%)	59 (8.4%)	150 (21.2%)	51 (7.2%)				
Richest	682	184 (27.0%)	56 (8.2%)	113 (16.6%)	58 (8.5%)				

**Table 2** gives a descriptive analysis of the prevalence of stunting, wasting, underweight, and overweight children by maternal, household, and child characteristics. The p-value is computed using a chi-square test where stunting, wasting, underweight, and overweight were compared to normal children in each group. *p_s_, p_w_, p_u_*, and *p_o_* represent the p-values for stunting, wasting, underweight, and overweight, respectively. **Table 2** shows the proportion of different children's statures by maternal statures and socioeconomic status. The percentage of normal children can be computed by subtracting the corresponding value at each group from 100. For example, the percentage of stunted children in the urban areas is 40.7 so the normal or non-stunted children are 59.3% (100–40.7%). We found that compared to mothers with higher education, mothers with lower education had a higher percentage of stunting, underweight, wasting, and overweight children. Likewise, mothers with short stature, residence in rural areas, and low socioeconomic status had a higher percentage of stunting, underweight, wasting, and overweight children.

**Figure 1.**
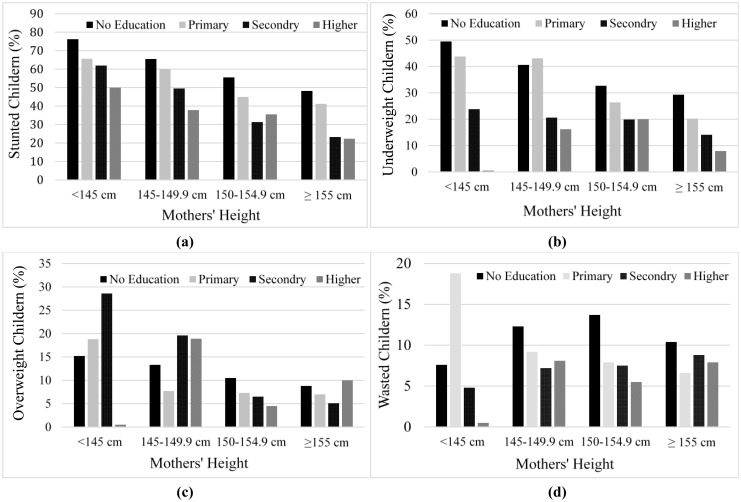
Prevalence of malnutrition children in PDHS 2012–13 by mothers' education and splitted-heights: (a) stunted children, (b) underweight children, (c) overweight children, and (d) wasted children.

Figures 1(a)–(d) show the percentage of stunted, underweight children, overweight children, and wasted children in Pakistan by mothers' education and height. We can see that short stature mothers with no education have a higher percentage of stunted and underweight children as compared to tall mothers with no education (Figure 1 (a) and (b)). However, no clear trend is observed in overweight and wasted children by mothers' education and height. Compared to tall stature mothers with higher education, short stature mothers with lower education have a higher percentage of stunted and underweight children. At each education level, short stature mothers are more likely to have stunted and underweight children. However, the percentage decreases as the education level increases. We did not find a clear trend for overweight and wasted children.

**Table 3** shows that compared to tall stature mothers, children born to short stature mothers are more likely to be stunted, underweight, and overweight. Likewise, compared to mothers with higher education, children of mothers with no education are more likely to be stunted, underweight, and wasted. The odds of affecting child malnutrition via maternal weight is minimal as the odds ratios are close to 1. The residence and wealth index are statistically significant, where a lower wealth index is associated with a higher odds ratio of stunting, underweight, and overweight. Child sex and child age were significant in the stunting and underweight models, and both were non-significant for overweight and wasting. Girls had approximately 20% higher odds of being stunted or overweight compared with boys. Moreover, we tested the interaction effect between maternal education and maternal height. None of these interactions, however, turned out to be significant and hence were discarded in the final models.

**Table 3.** Logistic regression models for stunted, underweight, overweight, and wasted children.

Variables	Reference Group		Stunting		Underweight		Overweight		Wasting	
			OR	CI	OR	CI	OR	CI	OR	CI
Mother height	Tall stature	Short stature	2.02**	1.67–2.45	1.48**	1.22–1.79	2.14**	1.63–2.79	0.93	0.69–1.24
Mother education	Higher	No	1.56*	1.16–2.09	2.56**	1.78–3.70	0.72	0.45–1.15	1.68*	1.03–2.75
		Primary	1.51*	1.11–2.05	2.19**	1.49–3.22	0.74	0.45–1.23	1.15	0.67–1.96
		Secondary	0.89	0.66–1.18	1.33	0.92–1.94	0.83	0.53–1.31	1.17	0.71–1.91
Mother's age		Age	1.01	0.99–1.01	0.99	0.98–1.00	1.01	0.99–1.04	1.01	0.99–1.03
Mother's weight		Weight	0.99**	0.98–1.00	1.00**	0.99–1.00	1.00**	1.00–1.01	0.99**	0.99–1.00
Residence	Rural	Urban	1.25*	1.05–1.49	0.95	0.78–1.15	1.74**	1.31–2.30	1.09	0.83–1.44
Wealth index	Richest	Poorest	2.89**	2.11–3.96	1.16	0.82–1.64	3.50**	2.11–5.77	1.02	0.62–1.67
		Poor	2.30**	1.71–3.09	1.07	0.77–1.49	1.82*	1.12–2.99	0.89	0.55–1.43
		Middle	1.80**	1.38–2.38	0.94	0.68–1.29	1.46	0.93–2.31	1.01	0.65–1.57
		Richer	1.47*	1.14–1.89	0.89	0.66–1.19	1.02	0.67–1.56	0.89	0.59–1.36
Child sex	Female	Male	1.21**	1.05–1.41	1.19*	1.02–1.39	1.03	0.82–1.31	1.19	0.95–1.51
Child age		Age	1.02**	1.02–1.03	1.00**	1.00–1.01	1.00	0.99–1.00	0.98**	0.97–0.99

Note: ** p ≤ 0.01, * p ≤ 0.05. OR = odds ratio, CI = confidence interval.

## 4. Discussions

This study revealed the potential determinants of stunting, underweight, wasting, and overweight (malnutrition indicators) among children under age five years in Pakistan. First, we found that mothers with short statures have a higher prevalence of stunting, wasted, underweight, and overweight children as compared to taller mothers. Second, we found that the higher prevalence of stunting, wasted, underweight, and overweight among under-five children were associated with mothers of low education level, poor socioeconomic status, and short maternal stature. We hypothesized that shorter mothers may be at a higher disadvantage and hence have more room to let the effect of education manifests. However, we did not find a significant interaction effect between maternal education and maternal stature on children's growth. This suggests that despite children born to mothers with shorter stature have more room for improvement, and maternal education has a positive effect on children's growth, effects of education, however, are similar across maternal statures. This puts children born to mothers with short statures at higher risk of poor stature even if the mothers are educated.

Sinha et al. [10] found that mothers with a height of less than 150 cm were about 2.3 times more likely to have a stunted child compared to mothers with heights greater than 150 cm. Dewey et al. [33] found that children of mothers with heights less than 145 cm were at higher risks of having stunted and underweight children compared to mothers with heights greater than 160 cm. Rachmi et al. [34] found that children from parents with short statures were more likely to be stunted and overweight. However, we found that maternal stature was not statistically significant in the wasting model. This could be because short stature mothers have a thinner pelvis that raises the probability of cephalopelvic disproportion and obstructed labor [33]. Moreover, biologically, since short stature mothers may have lower health stock, the available nutrients to the fetus may be insufficient, which could result in intrauterine growth restriction and low birth weight, influencing babies' health [13].

The regression analysis confirmed our first hypothesis that mothers with lower education had a higher percentage of stunting, underweight, and overweight children under five. Kumar et al. [35] found that compared to well-educated mothers, children of uneducated mothers have a higher number of undernourished children. Similarly, Kandala et al. [36] showed that mothers with no or with primary education have a higher proportion of stunted children compared to mothers with secondary or higher education. Similar results were found by a study that used the Sri Lankan DHS 2006–2007 [37]. We also found that mothers with short statures and low education levels have a higher number of stunted, underweight, and overweight children. This trend decreases when either the education level increases for a given maternal height or maternal height increases for a given education level. Our findings are similar to Wirth et al. [38], who found that maternal height and education were the most solid causes identified of stunting, and mothers of a normal body mass index are more likely to have non-stunted children [39].

We found that living in urban areas is a risk factor for stunting. It is possible that urban areas have higher living expenses where households find difficulties in managing their spending. This finding is consistent with a previous study [40]. Likewise, Dekker et al. [41] showed that mothers with poor wealth index have a higher percentage of malnourished children. The higher prevalence of undernourished children is because of poor socioeconomic status, and the inability to purchase nutritious food (even if they have the knowledge of it). The higher prevalence of overweight children in urban areas could be due to the feeding of under-nutritious (low in vitamins, minerals, and other healthy micronutrients) or junk food, which results in overweightness [42].

This study has limitations that need to be considered in interpreting the results. (1) This study is based on cross-sectional study design; thus, it is difficult to determine causal effects among the variables. (2) Data on micronutrient intake and supplementary nutritional factors directly linked to the dietary status of children are not available.

## 5. Conclusion

In Pakistan, a significant fraction of children under age five suffer from poor growth, and a substantial proportion of mothers have no or low education. While education does not have different marginal effects for mothers with different physical stature, it does play an essential role in elevating poor children's growth in Pakistan.
